# Growing cell walls show a gradient of elastic strain across their layers

**DOI:** 10.1093/jxb/ery237

**Published:** 2018-06-26

**Authors:** Marcin Lipowczan, Dorota Borowska-Wykręt, Sandra Natonik-Białoń, Dorota Kwiatkowska

**Affiliations:** Department of Biophysics and Morphogenesis of Plants, University of Silesia in Katowice, Katowice, Poland

**Keywords:** Buckling, *Helianthus annuus* hypocotyl, *Hordeum vulgare* coleoptile, mechanical heterogeneity, mechanical stress, primary cell wall, *Taraxacum officinale* peduncle, Young’s modulus

## Abstract

The relatively thick primary walls of epidermal and collenchyma cells often form waviness on the surface that faces the protoplast when they are released from the tensile in-plane stress that operates *in situ*. This waviness is a manifestation of buckling that results from the heterogeneity of the elastic strain across the wall. In this study, this heterogeneity was confirmed by the spontaneous bending of isolated wall fragments that were initially flat. We combined the empirical data on the formation of waviness in growing cell walls with computations of the buckled wall shapes. We chose cylindrical-shaped organs with a high degree of longitudinal tissue stress because in such organs the surface deformation that accompanies the removal of the stress is strongly anisotropic and leads to the formation of waviness in which wrinkles on the inner wall surface are always transverse to the organ axis. The computations showed that the strain heterogeneity results from individual or overlaid gradients of pre-stress and stiffness across the wall. The computed wall shapes depend on the assumed wall thickness and mechanical gradients. Thus, a quantitative analysis of the wall waviness that forms after stress removal can be used to assess the mechanical heterogeneity of the cell wall.

## Teaser

Plant cell walls are layered nanostructures that exhibit gradients of stiffness and tensile stress across their layers. The gradients are crucial for the regulation of cell wall growth and plant morphogenesis.

## Introduction

The walls of living plant cells in a turgid state are under tensile in-plane stress. Turgor pressure is the basic source of this stress (further referred to as turgor-driven stress): the cell walls are like the walls of a pressurized vessel. An additional source of stress in cell walls are tissue stresses, sometimes referred to as tissue tension. Their origin, either from differential growth or from non-uniform structural and mechanical properties, is still under debate (Peters and Tomos, 1996, 2000; Hejnowicz, 2011; Baskin and Jensen, 2013). Nevertheless, it is well documented that in the cylindrical-shaped shoot organs, such as hypocotyls or stems, the outer tissues are under tensile tissue stress in the longitudinal direction. This stress is superimposed on the turgor-driven stress (Hejnowicz and Sievers, 1995*a*). Tissue stresses exist only *in situ* and disappear when the tissue is isolated from the organ. Although tissue isolation does not affect turgor-driven stress, this stress can be removed by plasmolysis. The tensile in-plane stress in cell walls is necessary for growth, the irreversible deformation of the cell wall (Green, 1962; Dumais, 2013), and is a regulatory factor in plant development (Hamant, 2013). Therefore, knowledge of cell wall mechanics is the basis for understanding plant morphogenesis at both the cell and organ scales (Bidhendi and Geitmann, 2016).

It has been shown that the removal of tensile stress (both tissue and turgor-driven) from the relatively thick primary cell walls leads to the formation of waviness of the wall layers that face the protoplast in the epidermis and collenchyma of growing plant organs such as coleoptiles or hypocotyls (Hejnowicz and Borowska-Wykręt, 2005). The postulated mechanism of the formation of this waviness is Euler buckling. This is a reversible deformation that occurs when a critical value of the in-plane compressive force is surpassed, in the course of which an initially flat plate becomes sinusoidal. Buckling may also lead to change of a shell shape from smooth to sinusoidal or to the formation of wrinkles on a surface of a multi-layered shell (Timoshenko and Young, 1965; Ugural, 1999; Chen and Hutchinson, 2004; Sharon and Efrati, 2010). This type of buckling is unlike the irreversible local buckling in which a catastrophic ‘kink’ is formed (Romberger *et al.*, 1993).

In layered structures such as cell walls, the in-plane compressive force that is a prerequisite for buckling can originate from differences in the elastic strain of the layers. Hejnowicz and Borowska-Wykręt (2005) postulated that the strain heterogeneity in buckling cell walls is related to a gradient in tensile in-plane stress in the wall *in situ*; the innermost layer, being the youngest one, is the least stressed, while in the deeper layers the stress increases with the distance from the youngest layer. However, these authors provided only limited support for their postulate. First, they did not verify the existence of elastic strain heterogeneity of the wall layers. Second, they did not consider the effect that heterogeneity in wall stiffness may have on buckling. Moreover, the calculations that they presented in support of their postulate involved simplified equations to describe the buckling of a structure composed of a thin, stiff film attached to a thick substrate of a much lower stiffness (Gough *et al.*, 1940). When in-plane compression is applied to such a structure, the substrate shrinks more than the film and, as a result, the film buckles. Complex models dedicated to the buckling phenomenon also focus on this type of layered structure (Cerda and Mahadevan, 2003; Huang *et al.*, 2005; Sharon and Efrati, 2010). These models apply to diverse biological phenomena such as the wrinkling of a fruit surface during drying or the folding of the brain cortex during fetal development (Yin *et al.*, 2008; Chen and Yin, 2010). However, a structure composed of a thin, stiff film attached to a thick, soft substrate is not a realistic representation of the plant cell wall. The wall is structurally more homogeneous and the relative thickness of the wall portion that forms the waviness is much larger.

Therefore, in this work we readdress the postulate that buckling explains the formation of waviness of the cell wall layers after the removal of tensile in-plane stress. We first gathered empirical data on the deformation of cell walls due to the removal of the stress in different growing plant organs that have a cylindrical shape. Then, using the original computation protocol with assumptions that were compatible with the empirical data, we assessed the shapes of buckling wall layers and explored what conclusions on cell wall mechanics could be drawn from the buckling phenomenon. It was our intention to create a computation protocol that would be applicable to the cell wall that was simple and intuitive at the same time, so that the interpretation of computational and empirical results would be straightforward.

## Materials and methods

### Variables used to describe the shapes of cells or cell wall layers

The surface of cylindrical-shaped organs such as hypocotyls or coleoptiles is not smooth because it comprises the outer periclinal walls of numerous epidermal cells. We analysed the geometry of the epidermal surface by estimating the local curvature of these walls for individual cells, that is, the cell curvature. In order to compare the geometry of the epidermis *in situ* and after stress removal, we assessed the maximal and minimal cell curvatures of the epidermal surface (Dumais and Kwiatkowska, 2002).

When the tensile stress is removed from the outer tissues of coleoptiles or hypocotyls, the cell wall layers that face the protoplast undergo buckling, which leads to the formation of waviness. Such a change in the geometry of the cell wall layers can be also analysed by comparing the surface curvature. However, for our computations it was feasible to assess the shapes of the wall layers that underwent buckling by measuring the amplitude and wavelength of the waviness.

### Plant material and growth conditions

The experiments were performed on the elongating peduncles of blooming inflorescences of dandelion (*Taraxacum officinale*), etiolated hypocotyls of sunflower (*Helianthus annuus* cv. ‘Lech’), and etiolated coleoptiles of barley (*Hordeum vulgare* cv. ‘Stratus’). The dandelion plants were collected from pastures near Bielsko-Biała, southern Poland. The sunflower and barley plants were grown in a chamber. Sunflower achenes and barley caryopses were surface sterilized by immersion in 1% sodium hypochlorite for 20 min and then rinsed in tap water. After germinating on wet blotting paper for 24 h, the diaspores were transferred to plastic containers filled with moist vermiculite and grown in darkness at room temperature (23 °C). The sunflower hypocotyls were collected after 5 d when they were ~60–70 mm long; barley coleoptiles 40 mm long were collected after 4 d.

### Nomarski light microscopy

Epidermal strips, 5–10 mm long and ~1 mm wide, were peeled from the elongation zone of the sunflower hypocotyls, that is, the region 10–20 mm below the cotyledonary node. Strips from the barley coleoptiles, 5 mm long and 2 mm wide, were peeled from the region 5–10 mm below the coleoptile tip. Strips of a similar size were peeled from the dandelion peduncles from the region 20–25 mm below the capitulum. The strips (with the tissue stress removed), which contained at least one layer of cortical cells in addition to the epidermis, were immersed in a solution of 300 mM mannitol or 170 mM NaCl for 15–20 min, which caused incipient plasmolysis (to remove the turgor-driven stress). A microscope (Nikon Eclipse 80i) equipped with a Nomarski interference contrast system was used to examine the samples.

### Transmission electron microscopy

For transmission electron microscopy (TEM), epidermal strips with a circumferential length of ~4 mm and a width of 2 mm were peeled in the circumferential direction from the same hypocotyl, coleoptile, or peduncle regions as the strips that were used for Nomarski microscopy. A solution of 300 mM mannitol in 50 mM phosphate buffer (pH 7) was applied for 15 min to plasmolyse the samples. The samples were then immersed in fixative solution (2.5% glutaraldehyde, 2% caffeine) for 3 h, buffered in 300 mM mannitol in 50 mM phosphate buffer (pH 7) post-fixed with 1% OsO_4_ for 2 h, rinsed in water, and dehydrated in a graded ethanol series. After dehydration, the samples were embedded in Epon resin. Ultrathin longitudinal–radial sections (the plane across the periclinal walls and parallel to the long cell axis), 90 nm thick, were mounted on copper grids (200 mesh), double-stained with uranyl acetate and lead citrate, and examined by TEM (Hitachi H500).

### Isolation and observation of outer periclinal cell wall fragments

Deformation of an outer periclinal cell wall, which occurs due to the removal of stress by the isolation of a wall fragment, was examined in the etiolated hypocotyls of 4–5-d-old sunflower seedlings. First, a 3 mm thick disc (a cylindrical-shaped fragment) was excised from a hypocotyl ~10 mm below the cotyledons and immersed in one of three media: deionized water (hypotonic solution); 119 mM NaCl (isotonic solution with an osmolality of 238 mOsm/kg); or 170 mM NaCl (hypertonic solution). A strip was peeled from the disc immersed in the solution, in the circumferential direction. The strip, containing the epidermis and a single layer of the underlying parenchyma, was placed on a glass slide (with the epidermis facing the glass), one of its ends was immobilized using forceps, and the parenchyma together with the inner periclinal walls of some of the epidermal cells were removed with a razor blade. The epidermal fragment, including the exposed outer periclinal walls, was cut into longitudinal sections (along the hypocotyl axis) using a razor blade. The sections were observed in a drop of the respective solution under a microscope (Nikon Eclipse 80i) in the longitudinal–radial plane, that is, the same plane as the ultrathin sections.

### Assessment of the shape and thickness of cell walls

**Fig. 1.**
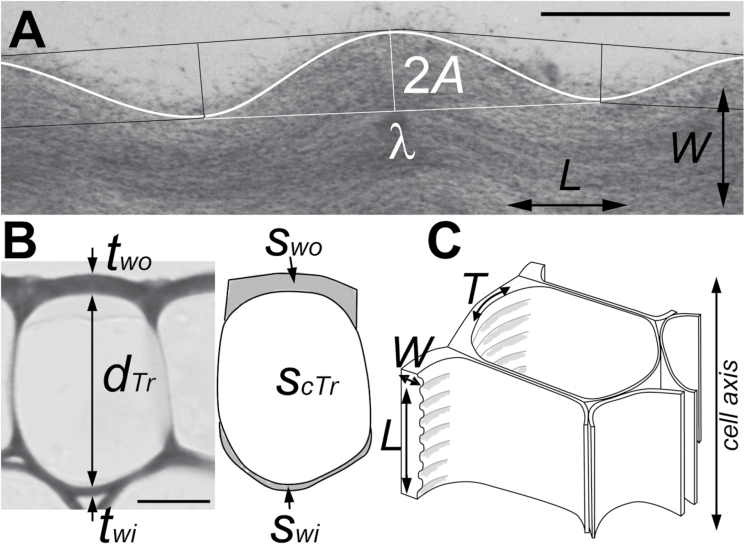
Cellular parameters used in computations. (A) Transmission electron micrograph showing a portion of radial–longitudinal ultrathin section of a cell wall fragment forming waviness. Lines superimposed on the micrograph were used to measure the wavelength (λ), amplitude (*A*), and the length of the most wavy layer (white curved line). Arrows indicate the directions *L* and *W* labelled in (C). Bar=0.5 µm. (B) Semithin cross-section (light microscopy, toluidine blue staining) of a sunflower epidermal cell. Parameters used for the assessment of in-plane wall stress are marked on the micrograph and its schematic representation (right): the thickness of the outer and inner periclinal cell walls (*t*_wo_ and *t*_wi_, respectively); the surface area of these walls (*s*_wo_ and *s*_wi_, respectively); and the height (*d*_Tr_) and surface area (*s*_cTr_) of the cell lumen. Mean ±SD values of the measurements for 10 cells are: *t*_wo_=2.2 ± 0.3 μm; *t*_wi_=0.5 ± 0.1 μm; *s*_wo_=36.4 ± 8.9 μm^2^; *s*_wi_=10.8 ± 3.6 μm^2^; *d*_Tr_=23.5 ± 2.0 μm; *s*_cTr_=293.9 ± 55.7 μm^2^. Bar=10 µm. (C) Diagram of part of an epidermal strip with waviness formed on the protoplast face of the outer periclinal walls of elongated epidermal cells; the long axis of the cells is in the direction *L*. Arrows indicate the three directions in the cell wall referred to in this paper: in-plane longitudinal (*L*); in-plane transverse (*T*); and across the wall (*W*).

Measurements were performed in the TEM micrographs of the longitudinal–radial sections of 12 cell wall fragments, obtained from epidermal strips of five sunflower hypocotyls, six wall fragments from four epidermal cells of one barley coleoptile, and three wall fragments from one dandelion peduncle (each fragment from a different cell). From each micrograph, the wavelength (λ) and amplitude (*A*) were assessed as the means of 4–20 measurements (Fig. 1A). The total wall thickness, the thickness of the wavy wall portion, and the lengths of the most wavy and non-wavy layers were estimated in the same images. Micrographs showing the cell wall sections with the most distinct layering were also used to estimate how the amplitude of the waviness of a layer changed with the distance of the layer from the straight cell wall portion. These measurements were made for four fragments of the sunflower cell walls, four of barley, and two of dandelion (each fragment from a different cell).

For the sunflower epidermis, various cellular parameters (Fig. 1B) were additionally measured in semithin cross-sections of 10 cells using ImageJ (https://imagej.nih.gov/ij/). These data were used to calculate the turgor-driven stress in the wall plane.

### *In vivo* replicas and scanning electron microscopy

For the sunflower hypocotyls and barley coleoptiles, changes in the shape of the epidermal surface due to the removal of stress were examined using the sequential replica method (Kwiatkowska and Burian, 2014). Two replicas (silicon polymer moulds) were taken from the surface of each portion of epidermis: *in situ* and from strips isolated and plasmolysed in 170 mM NaCl solution for 10–15 min. Casts obtained by filling the moulds with epoxy resin were sputter-coated and observed under a scanning electron microscope (SEM; Philips XL30 TMPESEN). Two images were obtained for each examined replica as a stereopair, tilted by 10º with respect to each other.

### Assessment of the surface strain of epidermal strips

Deformation due to stress removal was assessed for 11 sunflower epidermal strips, 15 barley strips, and 27 dandelion strips. To assess the strain in the longitudinal direction, the apical and basal edges of the epidermal portion that was to be peeled were labelled with dots 4.8–5.2 mm apart, using a waterproof marker, and photographed under a stereoscopic microscope (Nikon SMZ288). Next, the strip was peeled, plasmolysed as described above, and photographed again. To assess the strain in the transverse direction, two dots were marked along the organ circumference at a close distance (0.5–0.7 mm) in order to avoid measurement bias due to surface curvature (for hypocotyls the organ diameter was 1.8–2.5 mm, for coleoptiles 1.4–1.8 mm, and for peduncles 2.7–3.9 mm). The relative strain (shrinkage) in a given direction was computed as $100\%\frac{L_{k^{-}}L_{p}}{L_{k}}$

where *L*_k_ was the distance between the dots *in situ* and *L*_p_ was the distance after the stress removal.

### Quantification of cell curvature

The epidermal cell surface was reconstructed using the stereoscopic reconstruction protocol based on the stereopairs of SEM images (Routier-Kierzkowska and Kwiatkowska, 2008). A network of nearly isodiametric hexagons was overlaid on the reconstructed surface and the coordinates of their vertices were extracted (Ludynia, 2017). For each hexagon, the directions and values of the local maximal and minimal cell curvatures were computed for the surface by approximating the coordinates of the vertices belonging to the hexagon and its direct neighbours (Dumais and Kwiatkowska, 2002). The sets of local cell curvatures were compared for the individual cell walls before and after stress removal, in eight and seven portions of the sunflower and barley epidermis, respectively.

### Software used for computations, figures, and statistical analysis

All of the codes were written in Matlab (Mathworks, Natick, MA, USA). Statistical analysis, image processing, and artwork preparation were performed using Matlab, Adobe Design Premium CS4 (Adobe Systems Inc., USA) or CorelDRAW X6 (Corel Corp.).

## Results

### Empirical data on the cell and cell wall deformation due to stress removal

#### Assessment of in-plane stress in the cell wall in situ

First, we estimated the in-plane stress in the outer periclinal cell walls in the longitudinal and transverse directions *in situ* (*L* and *T* in Fig. 1C, respectively). This was done only for the epidermis of etiolated sunflower hypocotyls because both the turgor and tissue stresses are known for this tissue. Based on the measurements of the cellular parameters (Fig. 1B) and turgor pressure value, *P*=0.6 MPa, reported in the literature (Hejnowicz and Sievers, 1995*b*; Kutschera and Niklas, 2013), we assessed the turgor-driven stress in the longitudinal direction (σ_tL_) as 3.7 MPa and in the transverse direction (σ_tT_) as 5.2 MPa (see Supplementary Protocol S1 at *JXB* online). The total stress in the cell walls *in situ* is the sum of the turgor-driven and tissue stresses (Hejnowicz and Sievers, 1995*a*), which in the outer tissues of the sunflower hypocotyl is 2.5 MPa in the longitudinal direction and 0.06 MPa in the transverse direction (Hejnowicz, 1997). Thus, we predicted that the in-plane wall stress of the sunflower hypocotyl epidermis *in situ* is anisotropic, with the maximum in the longitudinal direction (σ_L_*≈*6 MPa) and the minimum in the transverse direction (σ_T_*≈*5 MPa). The examined epidermal or collenchyma cells of the dandelion and barley plants studied were elongated, similar to the epidermal cells of sunflower, and are also under tensile longitudinal tissue stress *in situ* (Hejnowicz, 1997; Niklas and Paolillo, 1998). Thus, we concluded that their walls were also under anisotropic tensile stress, with the maximum stress in the longitudinal direction.

#### Deformation of the epidermal strip surface after stress removal

Next, we assessed the surface strain of the epidermal strips that accompanied tissue isolation and plasmolysis. In all of the examined species, this strain was strongly anisotropic; the strips shrank on average by 7–17% in the longitudinal direction but by only 1–3% in the transverse direction (Table 1).

**Table 1.** Deformation due to stress removal and shape parameters of buckled wall portions

Parameter	Sunflower	Barley	Dandelion
Epidermal strips (analysed by stereoscopic microscopy)			
Longitudinal shrinkage (%)	12.0 ± 1.9 (11)	17.5 ± 1.8 (15)	6.9 ± 3.4 (30)
Transverse shrinkage (%)	0.9 ± 4.0 (4)	2.9 ± 1.7 (12)	2.1 ± 2.1 (7)
Cell wall sections (analysed by TEM)			
Longitudinal wall shrinkage calculated from difference in layer lengths (%)	6.2 ± 2.4 (12)	8.4 ± 2.2 (6)	4.1 ± 0.4 (3)
Cell wall thickness (µm)	1.67 ± 0.50 (12)	2.34 ± 0.26 (6)	2.53 ± 0.20 (3)
Thickness of wavy wall portion (µm)	1.10 ± 0.28 (12)	1.17 ± 0.15 (6)	1.41 ± 0.55 (3)
Amplitude (µm)	0.10 ± 0.03 (12)	0.14 ± 0.02 (6)	0.11 ± 0.01 (3)
Wavelength (µm)	1.52 ± 0.49 (12)	1.42 ± 0.18 (6)	1.70 ± 0.13 (3)
Wavelength/amplitude ratio	14.8 ± 3.1 (12)	10.0 ± 0.8 (6)	16 ± 0.9 (3)

Data are means of measurements ±SD; the number of samples is given in parentheses. Wall shape parameters measured in TEM images refer to outer periclinal walls of epidermis in sunflower hypocotyl or barley coleoptile, and of dandelion peduncle collenchyma.

Wall shrinkage was calculated from the difference in lengths of the straight outer layer (*L*_outer_) and the most wavy inner layer (*L*_inner_) measured in TEM micrographs, as $100\%\frac{L{\;}_{inner}-\;L_{\;outer}}{L_{\;inner}}.$

**Fig. 2.**
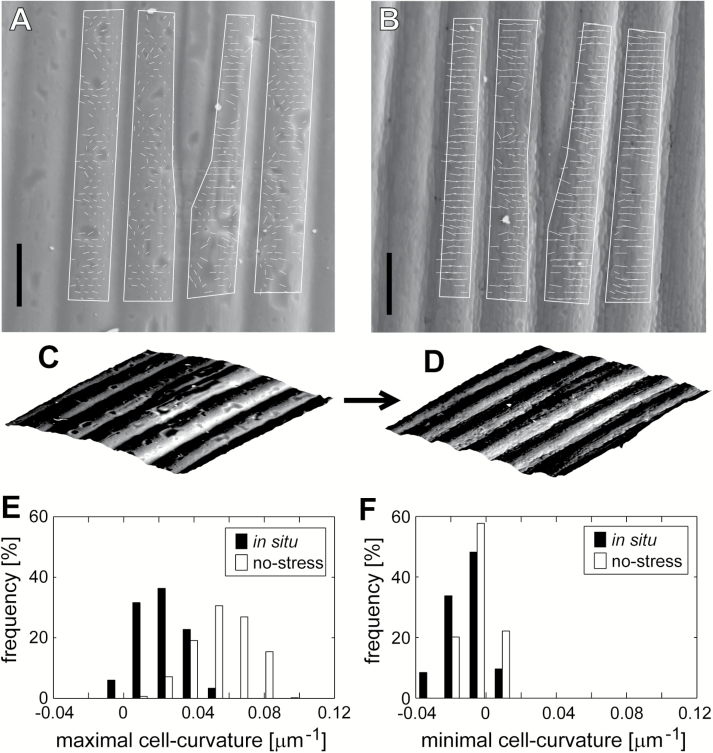
Changes in cell curvature of the outer periclinal cell wall of the epidermis of sunflower hypocotyl due to stress removal. (A, B) Scanning electron micrographs of replicas taken from the same part of the epidermis *in situ* (A) and after stress removal (B). Regions of the cell surface taken for cell curvature computation are outlined. Short lines within the outlined regions represent directions of maximal cell curvature; the line length is proportional to the cell curvature value. Note that the outlines do not provide landmarks for strain computation. Bars=20 µm. (C, D) Side views of example portions of the reconstructed surfaces shown in (A, B). (E, F) Histograms of maximal (E) and minimal (F) cell curvature values for the cell surface *in situ* and after stress removal, measured in the regions outlined in (A, B).

In sunflower and barley, the overall strip shrinkage was accompanied by changes in the shape of the outer periclinal walls of the epidermal cells (Fig. 2; Supplementary Fig. S1). Such changes in the cell curvature are important in estimating the local cell wall strain. For example, a wall that is initially convex in the transverse direction and flattens due to stress removal shrinks even more in this direction than the whole strip does. Thus, in order to estimate the strain at the scale of the periclinal cell wall rather than the whole strip, we had to assess changes in cell curvature. Both *in situ* and after stress removal, the direction of the maximal cell curvature in sunflower and barley was generally transverse to the long axis of the cell (Fig. 2A, B; Supplementary Fig. S1A, B), that is, the wall surface was convex (Fig. 2C, D; Supplementary Fig. S1C, D). In the direction of the minimal cell curvature, which was orthogonal to the maximal cell curvature direction, the surface was nearly flat both before and after stress removal (nearly zero cell curvature; Fig. 2F; Supplementary Fig. S1F). Surprisingly, after the stress removal, the outer periclinal walls did not flatten (Fig. 2D; Supplementary Fig. S1D). In sunflower, the walls became even more curved (Fig. 2E), that is, the maximal cell curvature increased significantly (*t*-test, *P<*0.01), while in barley, the change in the maximal cell curvature was less pronounced (Supplementary Fig. S1E). This means that in the transverse direction, the outer periclinal walls most likely shrink to a lesser extent than the whole strips. This probably happens because the strong longitudinal shrinkage leads to wall expansion in the transverse direction (as is described by the Poisson ratio), which contributes to the wall deformation.

To summarize, the strain of the examined periclinal walls was strongly anisotropic, stronger than the strain of the whole strips, with the maximal shrinkage in the longitudinal direction and nearly no shrinkage in the transverse direction.

#### Deformation of wall layers of the epidermal strip cells after the stress removal

**Fig. 3.**
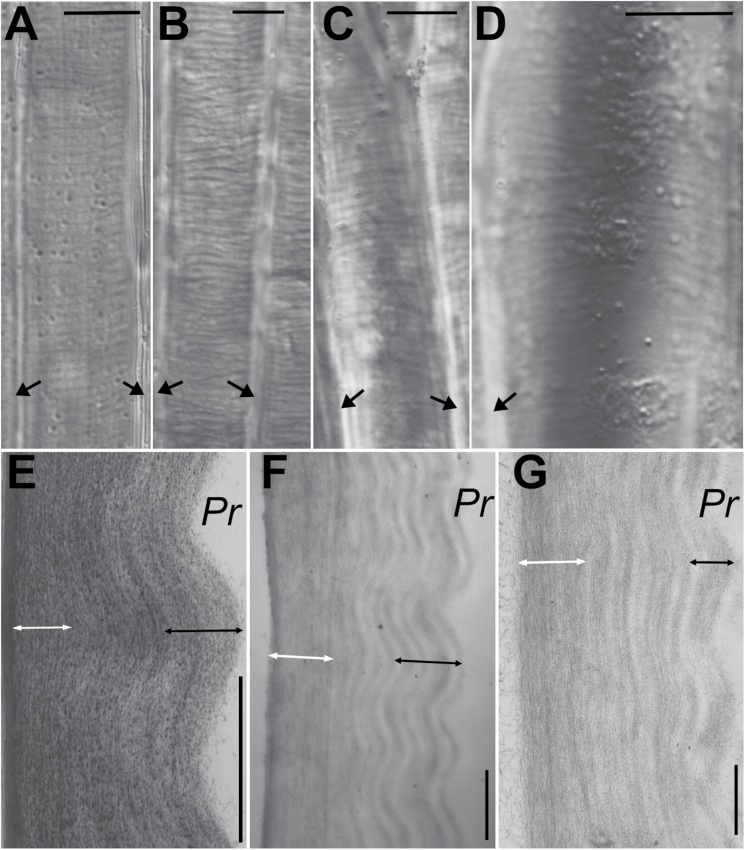
Cell wall waviness after stress removal. (A–D) Outer periclinal walls of sunflower hypocotyl epidermis (A), epidermis of barley coleoptile (B), epidermis of dandelion peduncle (C) and subepidermal collenchyma of dandelion peduncle (D), after isolation and plasmolysis of epidermal strips, observed by Nomarski microscopy. In these optical sections in the wall plane, the waviness appears as alternating light and dark bands. The long axis of the cells is vertical; black arrows mark the anticlinal walls, which are perpendicular to the image plane. Bars=10 µm. (E–G) Transmission electron micrographs of longitudinal–radial sections of sunflower epidermis (E), barley epidermis (F), and dandelion collenchyma (G). Wall fragments differ in the thickness of the straight wall portion (white arrows) and the portion with nearly uniform amplitude (black arrows). Pr, Protoplast face of the wall. Note that although the collenchyma walls of adjacent cells are attached by a middle lamella, unlike the superficial walls of epidermal cells, the visible collenchyma wall fragment in (G) belongs to one cell only because it became separated from the adjacent cell wall during tissue shrinkage. Bars=1 µm.

Next, we analysed the outer periclinal walls in the epidermal strips using the Nomarski microscope, which facilitates the visualization of any local variations in cell wall thickness. After the isolation and plasmolysis of the epidermal strips, alternating light and dark bands appeared in the plane of the cell walls when the focus was on the portion of wall facing the protoplast (Fig. 3A–D). The bands were always transverse with respect to the long cell axis. They resulted from the waviness of the inner wall layers, visible in TEM micrographs of the cell wall sections cut in the longitudinal–radial plane (Fig. 3E–G), which is orthogonal to the bands (plane *L*–*W* in Fig. 1C). In the outer periclinal walls of the subepidermal collenchyma or epidermal cells, the inner wall layers facing the protoplast were wavy, while the outer layers were not (Fig. 3E–G). The length of the straight wall layers measured in the TEM micrographs was smaller than the length of the most wavy layers by 4–8% on average (Table 1). Because all of the layers were straight prior to stress removal, this difference in length might suggest that after stress removal the outer wall layers that remain straight shrank in the longitudinal direction by 4–8% while the length of the inner wall layers remained unchanged. However, in all of the examined tissues, this assessed shrinkage was half the longitudinal shrinkage of the epidermal strip surface assessed under the stereoscopic microscope (Table 1). This difference can be explained by the longitudinal contraction of all the wall layers prior to buckling.

**Fig. 4.**
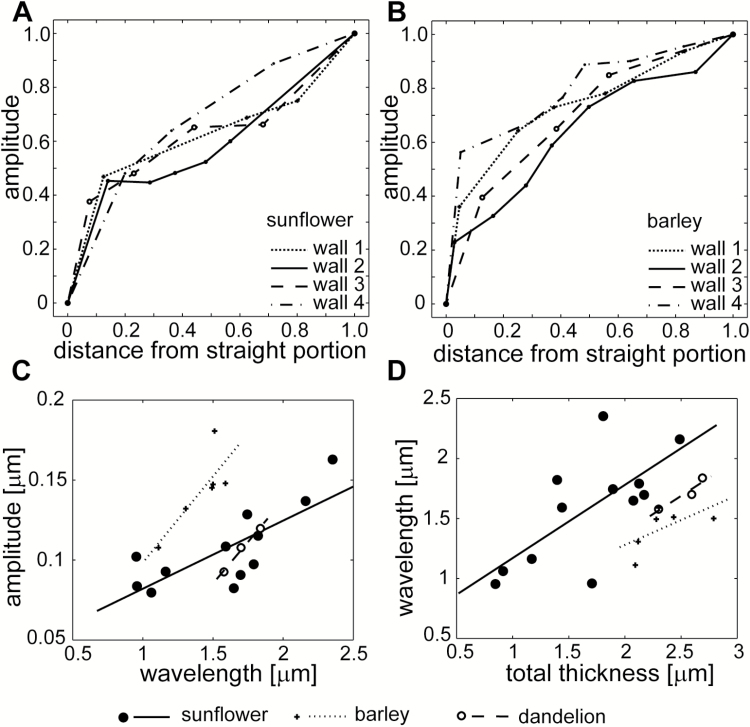
Variation of parameters characterizing cell wall waviness after stress removal. (A, B) Spatial variation of the amplitude of waves formed by layers of individual cell wall fragments in sunflower (A) and barley (B). In (A), wall 1 is the same as that shown in Fig. 3E; wall 4 in (B) is shown in Fig. 3F. All the amplitude and distance values were normalized (each value was divided by the maximal parameter value for the given cell wall). (C, D) Correlations between cell wall parameters. Lines are plotted based on linear regression analysis between the wavelength and amplitude of the innermost cell wall layers (sunflower: *y=*0.04*x+*0.04; *R*^*2*^*=*0.77; barley*: y=*0.11*x–*0.01; *R*^*2*^*=*0.65; dandelion: *y=*0.1*x–*0.07; *R*^*2*^*=*0.99), or between the total cell wall thickness and wavelength (sunflower: *y=*0.61*x+*0.56; *R*^*2*^*=*0.70; barley*: y=*0.45*x+*0.41; *R*^*2*^*=*0.36; dandelion*: y=*0.17*x+*0.61; *R*^*2*^*=*0.90).

The shape of the wavy wall surface resembled a cosine curve. Thus, we assumed that the mathematical function describing the waviness is *y*=*Acos*(*kx*), where *A* is the amplitude, $k\;=\;\frac{2\pi }{\lambda }$ is the wavenumber, λ is the wavelength (Fig. 1A), and *x* and *y* are coordinates in the *XY* system, where the *X* and *Y* axes are, respectively, in the directions *L* and *W* in Fig. 1A. The examination of individual sections showed that the wavelengths, and therefore also the wavenumbers, were the same for all of the wavy layers, and that the ‘waves’ formed by the layers of individual wall fragments were in the same phase (Fig. 3E–G). The amplitude increased with the distance from the straight wall layers, with the largest amplitude being observed on the inner wall surface facing the protoplast. It was, however, not proportional to the distance. Rather, the amplitude of the deeper layers changed quickly, while a relatively thick wall portion, which comprised many layers on the protoplast side (black arrows in Fig. 3E–G), exhibited a similar amplitude (Fig. 4A, B). The entire wavy portion of the wall was on average 1.1–1.4 µm thick depending on the tissue (Table 1); the mean amplitude of the inner wall surface varied from 0.10 to 0.14 µm, while the mean wavelength ranged from 1.4 to 1.7 µm. In all of the tissues, the wavelength was linearly related to the wave amplitude at the inner surface and, less strongly, to the wall thickness (Fig. 4C, D). The former correlation shows that a cosine curve was a good approximation of the wavy layer shape.

#### Deformation of cell wall fragments after isolation

**Fig. 5.**
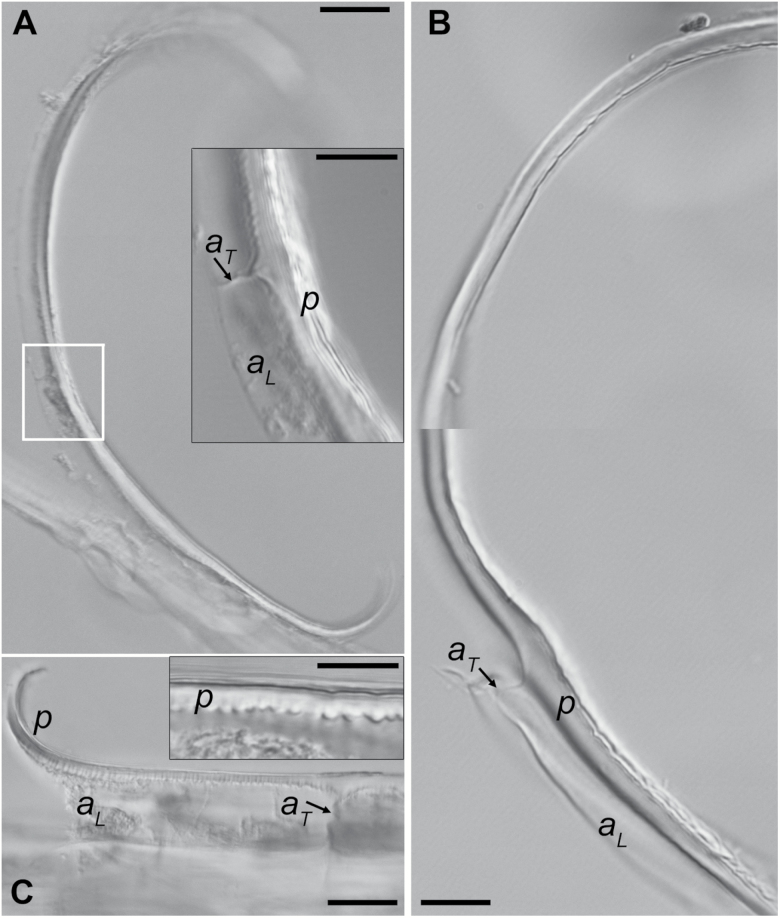
Deformation of isolated fragments of outer periclinal cell walls of sunflower hypocotyl. All the sections are shown in the longitudinal–radial plane, the same as the TEM sections in Fig. 3E–G. The fragments of outer periclinal wall (*p*), isolated in isotonic (A), hypotonic (B), or hypertonic (C) solution, bend outward from the organ surface. Note that fragments of the same wall that are attached to the longitudinal (*a*_L_) and transverse (*a*_T_) anticlinal walls remain nearly straight. The white frame in (A) indicates the wall fragment shown in the inset. The magnified inset in (C) shows waviness of the periclinal wall fragment that remains attached to the anticlinal walls. Bars=20 µm (A, C), 10 µm (B, insets in A and C).

A gradient of the elastic strain (shrinkage in the direction *L* shown in Fig. 1C after stress removal) across the cell wall (direction *W* in Fig. 1C) was a prerequisite for buckling. Therefore, we verified whether the layers of the outer periclinal wall of the epidermal cells shrink to different extents after the stress is removed, assuming that if this is the case, a wall fragment that is released from stress by being isolated (i.e. the periclinal wall is detached from anticlinal walls) should bend outward from the organ surface. In order to remove the stress, the epidermal wall fragments were isolated from discs that had been excised from sunflower hypocotyls and immersed in an iso-, hypo- ,or hypertonic solution. We expected that the bending of the wall fragments that had been isolated in hypo- or isotonic solution would be a direct effect of the differential shrinkage. In the case of the hypertonic solution, the bending would be driven by straightening of the buckled wall layers, when their margins ceased to be fixed by the neighbouring cell walls due to the isolation. We observed bending of the isolated wall fragments in all three solutions (Fig. 5), thus confirming the existence of an elastic strain gradient across the outer epidermal cell walls.

### Computational assessment of the cell wall waviness that appeared due to buckling

Next, using the original computation protocol, we explored how the cell wall mechanics affects the geometry of the wall waviness that is formed due to buckling. We considered various origins of the elastic strain gradient.

#### General assumptions

**Fig. 6.**
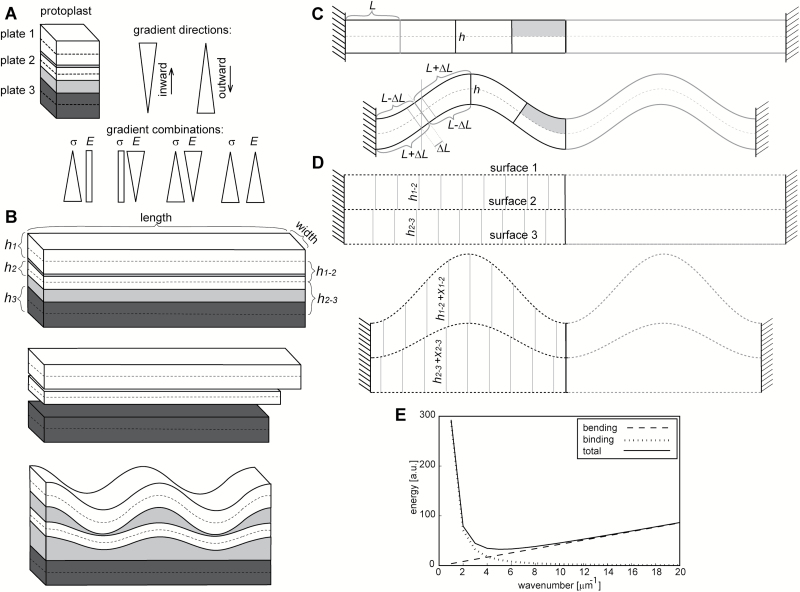
Assumptions used in the wall shape computation. (A) A cell wall fragment is represented by three plates embedded in an elastic medium (light grey). Plate 1 faces the protoplast. The gradients of in-plane modulus and pre-stress in the wall fragment are directed across the wall (direction *W* in Fig. 1C). In schematic representations of gradients, the highest values of in-plane modulus (*E*) or pre-stress (σ) are at the base of triangles, the lowest are at the apex. (B) *In situ* (upper panel), all the plates are straight. After stress removal, plates 1 and 2 (white) shrink to a lesser extent than plate 3 (dark grey), as shown in the middle panel. If all the plates are bound by the elastic medium, plates 1 and 2 buckle while plate 3 only shrinks (lower panel). For calculation of the energy required for stretching of the wall in the direction across the wall, plates are represented by surfaces (1, 2, and 3) marked with dashed lines in (A–D). (C, D) Upper panels refer to the wall *in situ*, i.e. under tensile stress; lower panels refer to the wall after stress removal. (C) The bending energy of plate 1 or 2 is computed for a plate part whose length equals the assumed wavelength divided into eight portions (an exemplary part of a plate, whose total length is twice the wavelength, is shaded) and multiplied by the wavenumber in order to obtain the energy for the whole plate. The plate ends are fixed. (D) The energy required for stretching in the direction across the cell wall is computed on the basis of the increase in distance between surfaces that represent the shapes of the three plates. All the symbols refer to parameters used in computations presented in Supplementary Protocol S3: distances between surfaces 1 and 2 (*h*_1-2_) and surfaces 2 and 3 (*h*_2-3_); increases in these distances after stress removal (*x*_1-2_ and *x*_2-3_, respectively); thickness of plates 1, 2, and 3 (*h*_1_, *h*_2_, *h*_3_); length of the plate portion, equal to one-quarter of the wavelength, taken for computation of bending energy (*L*) and its change due to bending (*ΔL*). (E) Example plot of energy components required for various wavenumbers, for the pre-stress gradient, where *h*_1_=1 μm; *E*_W_=0.5 *E*_2_; *E*_1,2,3_=72 MPa; σ_1_=0.29 MPa; σ_2_=3.74 MPa; σ_3_=7.2 MPa.

We assumed that the cell wall comprises three adherent portions that are composed of numerous layers. Each portion was represented by a plate that was embedded in an elastic medium (Fig. 6A). Because the amplitude of the layer waviness that is formed after stress removal is not uniform across the wall, the wavy wall layers were represented by two plates. Plate 1, which faced the protoplast, accounted for the wavy wall layers that were characterized by similar and high amplitudes. Plate 2, located deeper in the wall, represented the layers with amplitudes that decreased from high values to zero. Since the amplitude of the layers represented by plate 2 was more variable than that of plate 1, we assumed that the thickness of plate 2 (*h*_2_ in Fig. 6B) was half that of plate 1 (*h*_1_). The straight wall portion was represented by plate 3. The outward gradient of the shrinkage of the wall layers after stress removal (i.e. layer shrinkage increasing with the distance from the protoplast) was represented by differences in the elastic strain of plates, that is, their shrinkage in the longitudinal direction (Fig. 6B). Therefore, when the tensile stress was removed, plate 3, which shrank to the largest extent, put under compression plates 1 and 2, which shrank to a lesser extent. Because we regarded all of the plates as incompressible, plates 1 and 2 did not change in length under compression, but underwent buckling. The geometry of the wavy wall layers is periodic. Thus, we assumed that the plate ends were ‘fixed’ in terms of the boundary conditions used in mechanics (Fig. 6C). This assumption also accounts for the fact that we considered a wall fragment that was embedded in the periclinal wall of an individual cell.

Because the layers that build the wall are connected by the matrix, we also considered deformation in the direction across the wall (*W* in Fig. 1C), which corresponds to changes in the distances between the plates (*h*_1-2_ and *h*_2-3_ in Fig. 6D). Based on the expected differences between the cell wall stiffness (Cosgrove, 2016) in the direction across the wall (between layers of cellulose fibrils) and in the wall plane (parallel to the fibril orientation), we assumed that the stiffness across the wall would be either lower than or the same as the in-plane stiffness.

We set the boundary conditions on the strain, in-plane Young’s modulus, and the thickness of plates, as well as on the modulus in the direction across the wall, so that they were applicable to the cell walls (see Supplementary Protocol S2). We also made the following assumptions in order to simplify the computations. First, we assumed that all of the wall deformations that occurred after stress removal were reversible and that they observed Hooke’s law. This is supported by the observation that the cell wall waviness disappears after the recovery of turgor-driven stress due to deplasmolysis (Hejnowicz and Borowska-Wykręt, 2005). Second, we treated the cell wall as a planar structure that comprised plates instead of a quasi-cylindrical multilayered shell structure. This means that we neglected restraints due to the shape of the cell wall. However, the computations referred to a fragment of an individual cell wall that was virtually straight in the longitudinal direction (*L* in Fig. 1C) and slightly curved in the transverse direction (*T*). Third, when analysing the deformation of the elastic medium we ignored shear. This assumption did not affect the general trend but led to underestimation of the energy of the medium deformation (Landau and Lifshitz, 1959; Ugural, 1999). This effect was, however, counterbalanced by the assumption of a rather high modulus across the wall.

#### Computation of minimum energy configurations of plates

Heterogeneity in the elastic strain of layered structures can result from a gradient of the Young’s modulus or the opposite gradient of pre-stress (Cerda and Mahadevan, 2003; Hutchinson, 2013), the latter of which corresponds to the in-plane cell wall stress *in situ*. Therefore, we assumed that the strain gradient across the wall resulted from: (i) an outward pre-stress (σ) gradient (*σ*_1_ < *σ*_2_ < *σ*_3_); (ii) an inward modulus (*E*) gradient (*E*_1_ > *E*_2_ > *E*_3_ ), or (iii) two opposite gradients overlaid (Fig. 6A). Additionally, we considered the co-occurrence of two aligned gradients: (iv) the outward gradients of pre-stress (*σ*_1_ < *σ*_2_ < *σ*_3_) and the modulus (*E*_1_ < *E*_2_ < *E*_3_ ). In order to represent these four gradient combinations, we assigned various moduli and pre-stress values to each plate (see Supplementary Protocol S2). With these assumptions, we computed the strain (shrinkage) of each plate (ε_1,2,3_) that would occur when the stress was removed and selected the cases with the outward strain gradient (*ε*_1_ < *ε*_2_ < *ε*_3_) for further consideration.

Next, for each assumed set of the plate modulus and the pre-stress, we searched for the shapes of plates 1 and 2 that required the minimum energy necessary for deformation to occur due to the formation of waviness (Supplementary Protocol S3, Fig. S2). First, we identified the possible wavelengths and amplitudes of buckled plates 1 and 2 for a wide range of wavenumbers in the following manner. Taking a wavenumber from that range, we computed the corresponding wavelength (λ) and assumed that the length of plate 3 after stress removal was 2π µm, that is, the wavelength multiplied by the wavenumber. Because plate 3 did not buckle, this method ensured that the shapes of buckling plates 1 and 2 observed Euler’s law on buckling (Ugural, 1999). Keeping in mind that the length of all of the plates was the same before the stress was removed, and knowing the strains for each plate (ε_1,2,3_), we then computed the lengths of plates 1 and 2 after stress removal. Then, assuming λ and knowing the lengths of all of the plates, we used a numerical method to compute the amplitudes of buckling plates 1 and 2 (*A*_1_ and *A*_2_, respectively), searching for the length of the cosine function, which described the plate shape, that would fit the plate length. It should be noted that the lengths of the buckling plates were fixed and thus the amplitudes for various wavelengths differed. Second, for each set of λ, *A*_1_, and *A*_2_, we considered two energy components, one that was required to bend the plate (bending energy) and the other that was required to increase the distance between the plates (binding energy, which holds the plates together via an elastic medium).

In order to compute the bending energy component, we considered portions of a plate equal to one-quarter of the wavelength (Fig. 6C; see also Timoshenko and Young, 1965). Because of the symmetry of the cosine curve, the energy required to bend each portion is the same. Within each considered plate portion, there were two parts: a shortening part (grey in Fig. 6C) and an extending part; along the border of these two parts, the length does not change. The forces required to stretch one part are the opposite of those that are required to compress the adjacent part, but since they operate along the same distances (Δ*L* in Fig. 6C), the required energy is the same. Although the bending process is continuous, we simplified it by considering only 100 steps and computing the sum of the energy required for each step [we used the numerical solution of the total energy problem because it is unsolvable in an analytical way; see equation (12) in Supplementary Protocol S3].

For the same plate shapes, we computed the binding energy component required to increase the distance between the surfaces that represent the plates (*h*_1-2_ and *h*_2-3_ in Fig. 6D). For simplicity, only the deformation in the direction across the wall was considered. The binding energy is proportional to the product of Young’s modulus in the direction across the cell wall (*E*_w_) and the increase in volume (the surface area multiplied by the increase of thickness, i.e. *x*_1-2_ and *x*_2-3_ in Fig. 6D) between the surfaces that represent the plates, which accompanies the plate deformation [see equations (17–18) in Supplementary Protocol S3]. The energy components were multiplied so that they referred to the whole wall fragment.

The two energy components contribute to the total energy in a different way: the bending energy increases with an increasing wavenumber, while the binding energy decreases (Fig. 6E). We computed the total energy as the sum of these components, and searched for the minimal value in the wavenumber space.

#### Minimum energy configurations of the plates and the cell wall mechanics

**Fig. 7.**
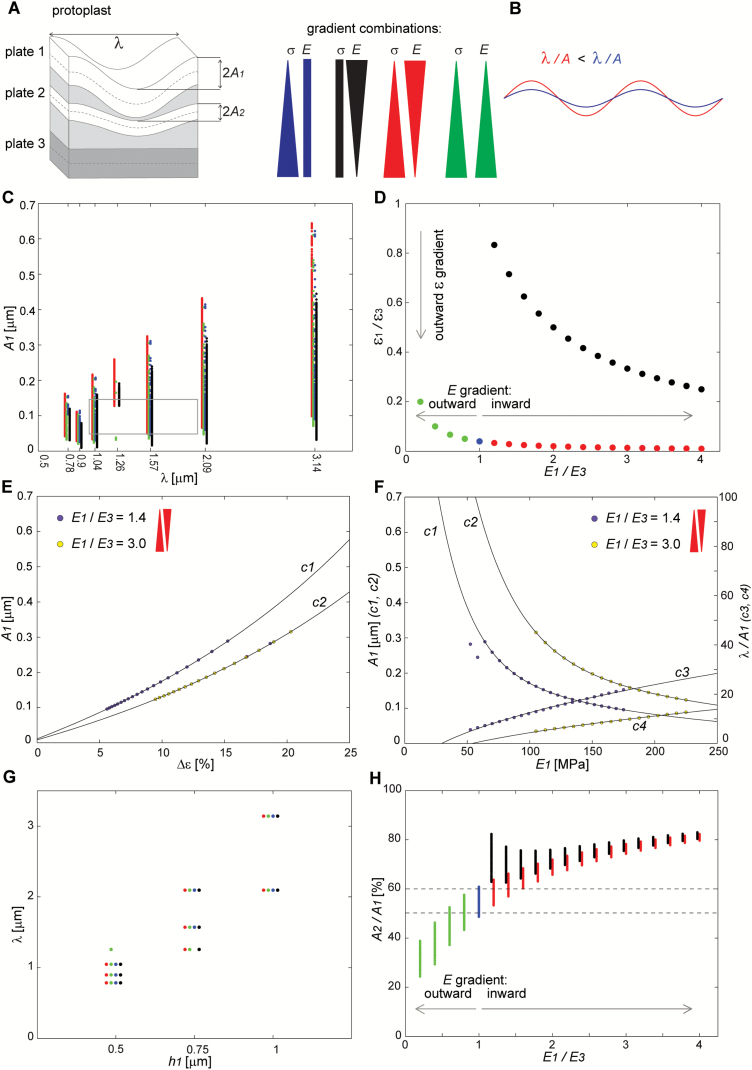
Response of the buckled plate shapes to the assumed cell wall parameters. (A) Parameters of the plate shape and the colour code that was used in the plots (C-H) in order to label the different gradient combinations: outward pre-stress gradient (blue); inward modulus gradient (black); outward pre-stress gradient overlaid by inward, i.e. opposite, modulus gradient (red); outward pre-stress overlaid by outward, i.e. aligned, modulus gradient (green). (B) Waviness characterized by different λ/*A*; the blue wave is ‘flattened’ compared to the red one. (C-H) Amplitudes and wavelengths of buckled plates for the assumed parameters. The amplitude and wavelength values within the range of empirical values are outlined in (C). Dashed lines delimit the range of the empirical values of *A*_2_/*A*_1_ in (H). The arrows in (D, H) indicate the directions of the increasing steepness of the modulus or strain gradient. The relationship between the shape and the mechanical parameters of plate 1 are plotted in (E, F) for the opposite gradients in the modulus and pre-stress; solutions for the other gradient combinations are presented in Fig. S4. The curves (*c1-4*) were fitted to the dots that represent the minimal energy solutions that were obtained for *h*_1_=0.75 μm; σ_3_=7.2 MPa; *E*_W_=0.5*E*_2_; and two values of *E*_1_/*E*_3_. Curves *c1, 2* in (E, F) are given by the equation $y\;=\;ae^{bx}+\;ce^{dx},$ where in (E): *a*>0; *b*<1; c, d ∈ (−1,0 ); in (F): *a*>1; b, d ∈ (−1,0);c ∈ (0,1). Each curve was fitted to the amplitude values that were related to the same wavelength. For curves *c3, 4* in (F) the equation is $y\;=\;a\sqrt{x\;}\;+\;b,$ where *a*>1; *b*<0. *R*^*2*^ >0.99 for all of the curves. In (C, G, H) the points that represent the different gradient combinations are offset laterally in order to improve visibility.

The computation results for all of the gradient combinations (see Fig. 7A) captured the geometry of the cell wall waviness that is formed after stress removal. The parameters that characterized the plate shapes of the minimum energy covered the range of the empirical data for the examined cell walls (parameter ranges for the innermost wall layer are framed in plots for plate 1; Fig. 7C; Supplementary Fig. S3A, B) such as wavelength, amplitude, or the wavelength/amplitude ratio (λ/*A*), higher values of which correspond to more flattened waviness (Fig. 7B).

A prerequisite for the formation of the waviness is the outward strain gradient in the wall *in situ*. Thus, we first examined the relationship between the strain gradient and the combined gradients of the modulus and pre-stress (Fig. 7A, D) for the boundary conditions that were dedicated to the cell wall. We characterized the steepness of the gradients by the ratios of the parameters that were assigned to plates 1 and 3. The pre-stress ratio, σ_1_/σ_3_, was either 1 (no gradient) or 0.04 (steep outward gradient). The modulus ratio, *E*_1_/*E*_3_, changed from 0.2 to 1 with decreasing steepness of the outward modulus gradient, and from 1 to 4 with increasing steepness of the inward gradient (arrows in Fig. 7D). The strain ratio, ε_1_/ε_3_, ranged from nearly 0 to 1, with the lower values corresponding to the steeper outward strain gradient. The outward pre-stress gradient alone (blue in Fig. 7A, D) resulted in a steep strain gradient. This effect was strengthened by the overlying of the opposite (i.e. inward), modulus gradient (red in Fig. 7A, D), but was weakened by the outward gradient of the modulus (green in Fig. 7A, D). The strain gradient that was generated by the inward modulus gradient alone (black in Fig. 7A, D) increased with *E*_1_/*E*_3_ but remained relatively weak. Thus, for the assumed boundary conditions, the steepness of the strain gradient increased with *E*_1_/*E*_3_, while the steep strain gradient was generated only with the contribution of the gradient of pre-stress.

The steepness of the strain gradient modulated the waviness by affecting the amplitude of the buckled plates—the amplitude of plate 1 (*A*_1_) increased non-linearly with the difference between the shrinkage of plates 3 and 1, $\Delta \varepsilon \;=\;\left|\varepsilon_{3}\;-\varepsilon_{1}\;\right|$ (Fig. 7E; Supplementary Fig. S4A–C). Thus, the steep strain gradient of the cell wall *in situ* was manifested by high amplitudes of the wavy wall layers after stress removal.

Next, we checked how the waviness responded to the resistance of plates to bending, which is related to the plate modulus and thickness, and to the resistance of the elastic medium to stretching (i.e. the modulus across the wall, *E*_w_). Lower amplitudes (e.g. of plate 1, *A*_1_; curves *c1* and *c2* in Fig 7F; Supplementary Fig. S4D–F) and the flattened waviness (higher λ/*A*; curves *c3* and *c4* in Fig. 7F; Supplementary Fig. S4D–F) were generated when the plate modulus was higher (non-linear relations). The wavelength was primarily sensitive to the plate thickness (*h*_1,2_): the thicker the plates, the bigger the wavelength (Fig. 7G) and the modulus across the wall (*E*_w_); for a lower *E*_w_ the wavelength decreased (Supplementary Fig. S3C; plots in Supplementary Fig. S3D–F show that the other parameters had no effect). Thus, the mechanical heterogeneity of the cell wall layers may be inferred from a quantitative analysis of their waviness. For example, if the waviness differs among cells from the same tissue, the stiffness of the recently formed cell wall layers is likely to be different, on the condition that the thickness of the wall portions with a similar amplitude is the same (the pre-stress had a minimal effect on the wave parameters; Supplementary Fig. S3D).

Ideally, the analysis of waviness of a cell wall should help to identify the mechanical gradient that existed in the wall prior to buckling. Indeed, the effect that the gradient combination had on the shape of the plates was differentiated by the ratio of the amplitudes of buckled plates 2 and 1 (*A*_2_/*A*_1_), which attained higher values for a similar waviness of these plates (Fig. 7H). As long as the pre-stress gradient was assumed, either alone (blue in Fig. 7H) or overlaid with the outward (green) or inward (red) modulus gradients, the amplitude ratio, *A*_2_/*A*_1_, increased with an increasing modulus ratio (*E*_1_/*E*_3_). The lowest *A*_2_/*A*_1_, that is, the most different shapes of plates 1 and 2, was for the aligned gradients of the pre-stress and the modulus (green in Fig. 7H). This counterintuitive result (the outward modulus gradient weakens the steepness of the strain gradient, and thus a higher rather than lower *A*_2_/*A*_1_ is expected) was the effect of the boundary conditions of the plate shrinkage. The highest *A*_2_/*A*_1_ was generated by the least steep inward modulus gradient (black in Fig. 7H; *E*_1_/*E*_3_ close to 1). When the steepness of this gradient increased (higher *E*_1_/*E*_3_), *A*_2_/*A*_1_ gradually converged with the *A*_2_/*A*_1_ that had been generated by the overlaid opposite gradients of the modulus and pre-stress (red in Fig. 7H). Thus, the *A*_2_/*A*_1_ is an emerging property of the mechanical heterogeneity of a cell wall that results from the interplay between the pre-stress and modulus gradients and the boundary conditions of the plate shrinkage.

We next attempted to infer which mechanical gradients were in the wall *in situ* by analysing changes in the amplitude across the cell wall layers. The amplitude of the layer that was located at one-quarter of the thickness of the wavy wall portion divided by the maximal amplitude corresponded to *A*_2_/*A*_1_. Thus, in the cell walls examined (Fig. 4A, B) with the amplitude at this location equal to 0.5–0.6 of the maximum, one could predict the outward pre-stress gradient overlaid by a weak modulus gradient, either inward or outward (delimited by dashed lines in Fig. 7H).

## Discussion

Cell wall stress, especially its anisotropy, affects the arrangement of the cortical microtubules and thus contributes to the well-documented growth regulation feedback (Landrein and Hamant, 2013; Sampathkumar *et al.*, 2014). However, the mechanism for sensing and transducing the anisotropic stress signal from the wall to microtubules that are located on the other side of the plasma membrane remains elusive (Hamant, 2013; Nick, 2013). The outward stress gradient in growing walls implies the existence of very low stress in the wall layers that are adjacent to the plasma membrane, even in elongating epidermal cells where the longitudinal tissue stress is high, which probably makes the strength of the stress signal similar to that in the protodermal cells of the shoot apices. On the other hand, the strength of the plasma membrane much lower than that of the wall (Wolfe and Steponkus, 1981), and proteins embedded in the plasma membrane are affected by small changes in its tension (Basu and Haswell, 2017). Therefore, if high stress in the youngest wall layer induced tension in the plasma membrane, the membrane would be damaged, whereas low absolute stress and minute changes in the stress and its anisotropy in the youngest wall layer could be sensed by the plasma membrane-embedded proteins and thus facilitate their contribution to the perception and transduction of cell wall stress. Such weak tensile stress in the youngest wall layer could be generated soon after its deposition—in the longitudinal direction by expanding older layers, and in the direction of the nascent cellulose microfibrils by their putative shortening (Alméras and Clair, 2016). The outward stress gradient also implies that cells may not regulate growth anisotropy simply by the microtubule-driven regulation of microfibril orientation in the youngest wall layer, because the older layers are subject to a higher stress. However, the relationship between growth anisotropy and microfibril orientation is more complex (Paolillo, 2000; Chan, 2012). In elongating epidermal cells, which have relatively thick outer periclinal walls, the inner walls may be important. Moreover, the anisotropy may also depend on the composition of the wall matrix (Saffer *et al.*, 2017).

Here, we demonstrate that in the growing cell walls of both dicots and grasses, which differ profoundly in the composition of their wall matrix (Fry, 2011), waviness is formed after the stress was removed, due to the buckling of the inner wall layers. A prerequisite for buckling is the heterogeneity of the elastic strain across the layers, which we confirmed by showing that the wall fragments bend spontaneously after being isolated. The strain heterogeneity is related to the mechanical heterogeneity of the cell wall. In order to gain insight into this phenomenon, we combined the empirical data on the formation of waviness with computations of buckled wall shapes. We chose cylindrical-shaped organs with tissue stresses because the surface deformation that accompanies stress removal is highly anisotropic and leads to the formation of waviness in which wrinkles on the inner wall surface are always transverse to the organ axis. This permitted simple computations, the interpretation of which was intuitive. The cell wall was represented by three relatively thick plates embedded in an elastic medium. We searched for the minimum energy configurations of buckled plates, considering the energy necessary to bend the plates and to stretch the medium. These energy components are also considered in models of the buckling of layered structures (Cerda and Mahadevan, 2003; Huang *et al.*, 2005; Hutchinson, 2013). The modelled structures, however, comprise a thin, stiff film on a thick, compliant medium that is far from representative of plant cell walls (see Supplementary Protocol S4 and Table S1 for a detailed comparison).

Our computations imply that waviness similar to that of the growing cell wall is generated by the outward pre-stress gradient, alone or overlaid by outward or inward stiffness gradients. The outward gradients of pre-stress and stiffness are expected in growing walls, but the occurrence of the inward stiffness gradient, in which the youngest wall layers are the stiffest, is disputable. The wall stiffness depends on a strain stiffening effect that may occur during wall deformation (Abasolo *et al.*, 2009; Kierzkowski *et al.*, 2012; Routier-Kierzkowska and Smith, 2013). In the case of fast-growing tissues, if strain stiffening indeed occurs, it would be more likely in older rather than younger wall layers. An inward stiffness gradient may, nevertheless, exist in the walls of cells that have ceased growing.

Buckling occurs when the threshold of compressive force is surpassed. The cell wall layers most likely shrink to some extent under compression before buckling. This explains why the extent of layer shrinkage that was assessed from the TEM micrographs was lower than the longitudinal shrinkage of the epidermal strips in all of the examined tissues. However, because the compression threshold of the primary cell wall layers is unknown, in our computations we assumed that the plates buckle as soon as the compression occurs. Nevertheless, the heterogeneity of the compression threshold across the wall likely influences the formation of wall waviness and may explain why the relatively thick inner portions of the examined walls, which comprised numerous wall layers, formed waviness of similar amplitudes. In a growing cell, new wall layers are not expected to be under tension because of the mode in which the new wall material, especially the cellulose microfibrils, is deposited. Rather, the tensile in-plane stress and the related elastic strain increase with the age of a layer up to a certain maximal value—that is, the further the layer is from the protoplast, the more it is stretched. The reason why numerous inner wall layers form similar waviness despite the expected stress gradient may be that they differ in their compression threshold, and therefore prior to buckling the inner layers shrink more under compression than the slightly older ones. In composite materials or nanostructures such as cell walls (Zhang *et al.*, 2016), the compression threshold is related to the orientation of reinforcing fibres and the mechanical properties of the matrix (Vincent, 1990; Schulgasser and Witztum, 1997). In particular, unidirectional fibre composites are prone to buckling under compression in the direction parallel to the fibres. The structure of the examined relatively thick primary cell walls is likely to be cross-polylamellate (in each layer the microfibrils are aligned but their orientation in adjacent layers differs), like that in the thick primary epidermal walls of the maize coleoptile or *Cucumis sativus* hypocotyl (Zhang *et al.*, 2016). Variation in the compression threshold may be related to such a spatial variation in reinforcement together with microfibril bundling or possible movements, and the heterogeneous composition and stiffness of the wall matrix (Bashline *et al.*, 2014; Zhang *et al.*, 2016, 2017; Majda *et al.*, 2017).

In conclusion, our experiments and computations prove the existence of an elastic strain gradient across the wall layers, with the youngest layers being the least stretched. This gradient is likely related to the deposition history of the wall layers and their modification after deposition. We show that a quantitative analysis of the wall waviness that arises after stress removal can be used to assess the mechanical heterogeneity of a wall *in situ*. The strain heterogeneity of the cell walls results from the outward gradient of pre-stress, which is likely accompanied by the Young’s modulus gradient in the same direction. Such a heterogeneous pre-stress may have significant implications for our understanding of the involvement of cell wall mechanics in regulating growth.

## Supplementary data

Supplementary data are available at *JXB* online.

Protocol S1. Estimation of turgor-driven in-plane wall stress.

Protocol S2 Specification of parameters used to compute the energy.

Protocol S3. Protocols used to compute the minimum energy configurations of plates.

Protocol S4. Comparison of models of buckling structures and computation protocol dedicated to cell walls.

Table S1. Wavelengths of buckled surfaces computed with the same assumptions using the computation protocol dedicated to cell walls and models on buckling of the layered structure.

Fig. S1. Changes in cell curvature of the outer periclinal cell walls of epidermis of barley coleoptile due to stress removal.

Fig. S2. Estimation of plate deformation during buckling.

Fig. S3. Minimum energy configurations of plate 1 computed for different mechanical parameters.

Fig. S4. Response of buckled plate shapes to plate mechanics

Supplementary Protocols and Table

Supplementary Figures
