# Erratum: Growing cell walls show a gradient of elastic strain across their layers

**DOI:** 10.1093/jxb/ery280

**Published:** 2018-08-09

**Authors:** Marcin Lipowczan, Dorota Borowska-Wykręt, Sandra Natonik-Białoń, Dorota Kwiatkowska

**Affiliations:** Department of Biophysics and Morphogenesis of Plants, University of Silesia in Katowice, Jagiellońska, Katowice, Poland

*Journal of Experimental Botany*, Advance Access publication 26 June 2018, doi:10.1093/jxb/ery237

The original online version of this article contained errors in the typesetting of an equation, listed under ‘Computation of minimum energy configurations of plates’. This equation, concerning the inward modulus (*E*) gradient described in part (ii), has now been corrected to read “(*E*_1_ > *E*_2_ > *E*_3_)”.

The publisher would like to apologise for this error.

