# Supplementary material for: Growing cell walls show a gradient of elastic strain across their layers
Source: J Exp Bot. 2018 Jun 26;69(18):4349–62. doi: 10.1093/jxb/ery237 (PMC6093493; doi:10.1093/jxb/ery237)
Supplement: Supplementary Protocols and Table [file ery237_suppl_supplementary_protocols_table_s1.pdf]

Supplementary Protocol S1. Estimation of turgor-driven in-plane wall stress

From definition:

(1) $$\sigma = \frac{F}{S}$$

where: σ – stress; F – force; S – surface area.

The force in the wall plane that is driven by turgor can be computed as:

(2) $$F = PS_c$$

where S_c is the surface area of the section of the cell lumen in the plane that is perpendicular to the force direction; P – turgor.

Thus, the in-plane turgor-driven stress in the cell wall is:

(3) $$\sigma_t = \frac{PS_c}{S_w}$$

where S_w is the surface area of the wall in the same section as that of the cell lumen.

Accordingly the in-plane stress in the transverse direction (direction *T* in Fig. 1C) is:

(4) $$\sigma_{tT} = \frac{PS_{cLn}}{S_{wLn}} = \frac{Pd_{Ln}d_{Tr}}{d_{Ln}(t_{wo}+t_{wi})} = \frac{Pd_{Tr}}{t_{wo}+t_{wi}}$$

and in the longitudinal direction (*L* in Fig. 1C):

(5) $$\sigma_{tL} = \frac{PS_{cTr}}{S_{wTr}}$$

where: S_{cTr} and S_{cLn} – the surface area of the cell lumen measured in the transverse (Tr) and longitudinal (Ln) sections, respectively; d_{Tr} and d_{Ln} – the height of the cell lumen in the transverse and longitudinal sections; S_{wTr} and S_{wLn} – the surface areas of the cell walls measured in the transverse and longitudinal sections ($S_{wTr} = S_{wo} + S_{wi}$ shown in Fig. 1B); t_{wo} and t_{wi} – the thicknesses of the outer and inner periclinal walls, respectively, measured in the transverse section (Fig. 1B). Because the anticlinal walls are very thin, their thickness and surface area are ignored.

Supplementary Protocol S2. Specification of parameters used to compute the energy

The minimal energy required for the deformation of the cell wall due to buckling was calculated for the sets of parameters, including the in-plane pre-stress and the Young's modulus of each plate, the Young's modulus across the wall, and the thickness of plates, which are specified in the following way.

In-plane pre-stress of plates The magnitude of the in-plane cell wall stress *in situ* is expected to vary because it depends on the cell geometry, mechanical wall properties and tissue stress. Thus, we considered three different maximal stress values: 5.2, 7.2 and 9.2 MPa. If the pre-stress gradient was assumed, one of these values was assigned to plate 3 (σ_3), while the pre-stress of plate 2 was $\sigma_2 = 0.52\,\sigma_3$ and that of plate 1 was $\sigma_1 = 0.04\,\sigma_3$. If there was no pre-stress gradient, the pre-stress of all of the plates was 5.2, 7.2, or 9.2 MPa. In such cases, the inward gradient of the in-plane Young's modulus of the plates was assumed to account for the elastic strain gradient across the wall.

In-plane Young's modulus of the plates Since very different values of the Young's modulus of growing cell walls have been reported in the literature (Lipchinsky *et al.*, 2013; Tittmann and Xi, 2014; reviewed in Cosgrove, 2016), we considered the moduli between 20 and 250 MPa. Modulus values from the range 25-150 MPa (with a step of 5 MPa) were assigned to plate 2. Then, the moduli for plates 1 and 3 were assigned so that $E_2 = \frac{E_1+E_3}{2}$ and the ratio $\frac{E_1}{E_3}$ ranged from 0.2 to 4 (with a step of 0.2).

From these pre-stress and modulus, the strain was calculated for each plate using Hooke's law. The parameters were further considered on the condition that **the strain (shrinking) of plate 3** was between 3 and 21% (the range was based on empirical data; Table 1) and that plate 3 shrank to the largest extent, while plate 1 shrank to the lowest extent.

Young's modulus in the direction across the wall We considered two different values of the Young's modulus in the direction across the cell wall (E_W; in direction W in Fig. 1C): the same as the in-plane modulus of plate 2, i.e. $E_W = E_2$ or half of this value, i.e. $E_W = 0.5\,E_2$.

Plate dimensions We considered a cell wall fragment comprising the three plates that were embedded in the elastic medium of a fixed width 10 μm (dimension in direction T in

Fig. 1C), i.e. not exceeding the real cell width (Fig. 3A-D). The length of the wall fragment (dimension in direction *L* in Fig. 1C) was such that it attained 2π μm after shrinking due to the stress removal so that the Euler law for buckling was observed. Because the walls varied in thickness and the contribution of the portions that formed the waviness after the stress removal, we considered three values of the thickness of wavy portion of the wall: 1, 1.5 and 2 μm (means for the examined species are within this range; Table 1). The corresponding thicknesses of plate 1 were 0.5, 0.75 and 1 μm, while the thickness of plate 2 was always half of the thickness of plate 1. The thickness of plate 3 did not affect the computation results (see *Protocol 3*).

References

Cosgrove DJ. 2016. Plant cell wall extensibility: connecting plant cell growth with cell wall structure, mechanics and the action of wall modifying enzymes. Journal of Experimental Botany **67,** 463–476.

Lipchinsky A, Sharova EI, Medvedev SV. 2013. Elastic properties of the growth-controlling outer cell walls of maize coleoptile epidermis. Acta Physiologiae Plantarum **35,** 2183-2191.

Tittmann BR, Xi, X. 2014. Imaging and quantitative data acquisition of biological cell walls with Atomic Force Microscopy and Scanning Acoustic Microscopy. In: Mendez-Vilas A, ed. *Microscopy: Advances in Research and Education*. Spain: Formatex Research Center, 161-172.

Supplementary Protocol S3. Protocols used to compute the minimum energy configurations of plates

All the deformations were assumed to observe Hooke's law:

(1) $$\sigma = E\varepsilon$$

where: ε – relative deformation (elongation/shrinking); E – Young's modulus.

From definition:

(2) $$\varepsilon = \frac{\Delta L}{L}$$

where: ΔL – the change (decrease/increase) in length; L – the length before deformation.

Thus from equation (1) of *Supplementary Protocol S1* and (1-2):

(3) $$F = ES\varepsilon$$

(4) $$F = ES\frac{\Delta L}{L}$$

In order to assess the **energy required for bending** of plates 1 and 2, we first calculated the energy for a plate of such a length that after bending (buckling) it attained the wavelength corresponding to the assumed wavenumber. In such a bending plate, extending or shrinking of plate portions, which are located on opposite sides of the neutral surface, requires the same work. Knowing this and that the cosine curve is symmetrical, we computed the energy for one of eight plate portions of length L and thickness *h/2* (like the grey portion in Figs 6C; S2) in the following way.

The shape of the wave is given by a function (Fig. S2):

(5) $$y = -a\cos(kx)$$

where: k – the wavenumber; a – amplitude. Thus:

(6) $$\tan\alpha = y' = ak\sin(kx)$$

Because we performed the calculations for $kx = \frac{\pi}{2}$ (Fig. S2)

(7) $$\tan\alpha = ak \qquad \text{and so} \qquad \alpha = \tan^{-1}(ak)$$

Thus from the triangle marked in blue in Fig. S2, *ΔL* can be calculated as:

(8) $$\Delta L = \frac{h}{2}\sin\alpha$$

So from (4) the bending force (F) for the considered plate portion is:

(9) $$F = ES\frac{h}{2L}\sin(\tan^{-1}(ak))$$

Thus the bending force for the considered portion of plate 1 is:

(10) $$F_1 = E_1 S_1 \frac{h_1}{2L_1}\sin(\tan^{-1}(a_1 k))$$

where: E_1 – the in-plane Young's modulus of plate 1; S_1 – half of surface area of the plate 1 cross section; h_1 – plate 1 thickness; L_1 – initial length of the considered plate 1 portion; a_1 – amplitude. The force is calculated for values of a_1 increasing gradually (in 100 steps) up to A_1 (final amplitude of plate 1 after buckling) so that changes of the plate shape during bending are accounted for.

From definition the work (W) is:

(11) $$W = \int F ds$$

where s is the distance along which the force operates. So the work required for bending of one portion of plate 1 is:

(12) $$W_1 = E_1 S_1 \frac{h_1}{2L_1} \int_0^{A_1} \sin(\tan^{-1}(a_1 k))\, da_1$$

This equation is solved numerically.

Because a buckled plate of the length equal to the wavelength comprises eight such portions the obtained value is multiplied by eight. Next, it is multiplied by the wavenumber, so that the computed energy refers to bending of the cell wall fragment, the length of which after buckling is 2π (corresponding to wavenumber equal to 1).

The same procedure was repeated for plate 2 and the results were summed up.

Note that when the bending energy is computed for different wavenumbers the amplitude has to change because we assume that the lengths of plates are constant. With such assumptions the bending energy is linearly related to the wavenumber (dashed line in Fig. 6E).

Next we computed the **energy necessary for stretching (binding energy)**, i.e. to increase the distance between surfaces representing the plates (dashed lines in Fig. 6B). This was computed for the cell wall fragment, the length of which was 2π after buckling. The stretching forces between surfaces 1 and 2 (F_{W1-2}), representing plates 1 and 2 and between surfaces 2 and 3 (F_{W2-3}) were considered. From (4) the forces between surfaces 1 and 2 are:

(13) $$F_{W1-2} = E_W S_W \frac{x_{1-2}}{h_{1-2}}$$

and between surfaces 2 and 3:

(14) $$F_{W2-3} = E_W S_W \frac{x_{2-3}}{h_{2-3}}$$

where: E_W – Young's modulus across the cell wall; S_W – surface area of the plate (normal to the direction across the wall); h_{1-2} and h_{2-3} – initial distances between surfaces 1 and 2 and 2 and 3, respectively; x_{1-2} and x_{2-3} – increase in these distances due to buckling (Fig. 6D).

From (11) and (13-14) the work necessary to stretch the medium between the surfaces 1 and 2 (W_{W1-2}) and 2 and 3 (W_{W2-3}) was calculated as:

(15) $$W_{W1-2} = \int E_W S_W \frac{x_{1-2}}{h_{1-2}} dx_{1-2} = E_W S_W \frac{x_{1-2}^2}{2h_{1-2}}$$

(16) $$W_{W2-3} = \int E_W S_W \frac{x_{2-3}}{h_{2-3}} dx_{2-3} = E_W S_W \frac{x_{2-3}^2}{2h_{2-3}}$$

The increase in distances between surfaces 1 and 2 (x_{1-2}) and 2 and 3 (x_{2-3}) were calculated as means for 1000 points, thus the works are:

(17) $$W_{W1-2} = \frac{E_W S_W}{2h_{1-2}} \left(\sum_{j=1}^{1000} \frac{-A_1 \cos kx_j + A_1 - (-A_2 \cos kx_j + A_2)}{1000} \right)^2$$

(18) $$W_{W2-3} = \frac{E_W S_W}{2h_{2-3}} \left(\sum_{j=1}^{1000} \frac{-A_2 \cos kx_j + A_2}{1000} \right)^2$$

where A_1 and A_2 are the final amplitudes of plates 1 and 2 after buckling. The energy required to stretch the cell wall in direction across the wall was calculated as the sum of W_{W1-2} and W_{W2-3}.

The **total energy** for the given wavenumber was calculated as the sum of the components necessary for bending and stretching.

This procedure was performed for the wavenumbers ranging from 1 to 20 μm^{-1} (with the step of 1 μm^{-1}). Based on these calculations the wavenumber for which the total energy attained the minimum was found (Fig. 6E).

Supplementary Protocol S4. Comparison of models of buckling structures and computation protocol dedicated to cell walls

The computation protocol presented in this manuscript is on purpose simplistic unlike complex models on buckling of layered structures. Moreover, our objective was to obtain computation results based on assumptions and boundary conditions applicable to the cell wall. Therefore, there is a fundamental difference in the assumptions between the models and our protocol. The models deal with layered structures comprising a thin film bond to a thick (in some cases semi-infinite) substrate. In our protocol, instead of the film we considered a relatively thick plate 1, which is the most wavy part of the structure after buckling. Plate 2 and the elastic medium in which the plates are embedded may be regarded as analogous to the portion of substrate used in the models that is stretched due to the buckling of the film. Plate 3 can be regarded as the part that is only shrinking under compression. To obtain results that are applicable to buckling cell walls, we assumed that plate 1 thickness is the same as that of the portion corresponding to "the substrate" (represented by plate 2, the thickness of which is half of the plate 1 thickness, embedded in the elastic medium). Such dimensions are very far from those assumed in the buckling models. Another important difference in assumptions between the models and our protocol is in stiffness. Most of the models assume that the film is at least ten times stiffer than the substrate (e.g. Cerda and Mahadevan, 2003). In our computations performed for sets of parameters comparable with cell walls, the Young's modulus of plate 1 is at most 2.5 times higher than modulus of plate 2 or the modulus across the wall, both of which correspond to the substrate. In some models it is assumed that an un-stretched film is bond to a pre-stretch substrate (e.g. Hutchinson, 2013). However, also then the film is taken as much thinner than the substrate.

The models show that if no pre-stretch of the substrate is assumed, buckling takes place only when the film stiffness is higher than that of the substrate. Such cases are also considered in our computations. Both the modelling and our computation results show that the two energy components, bending and binding (stretching) energy, behave differently in relation to wavelength. The energy required to stretch the substrate increases for increasing wavelength while the energy required for film bending increases when the wavelength decreases (e.g. Cerda and Mahadevan, 2003). Huang *et al.* (2005) model on buckling of structures comprising stiff film bonded to a compliant substrate, which is in turn bonded to a rigid support, shows that for the anisotropic strain, buckling leads to formation of sinusoidal wrinkles arranged in herringbone arrays or stripes. The latter pattern resembles that observed

in the cell wall. Thus, the modelling provides theoretical support for our computations focused on buckling of the cell wall.

We attempted to compare the results of the modelling and our computation protocol by applying the protocol with the assumptions that plate 1 represents the film while plate 2 and the surrounding elastic medium represent the substrate, and the thickness and stiffness of the plates are similar to those in buckling models (Table S1). The computed wavelengths were compared with the wavelengths that were obtained with the same assumptions from the equations that were used in the models on the buckling of layered structures in which the substrate thickness was either set (columns 4, 5; "thin-substrate limit") or semi-infinite (column 6; "thick-substrate limit" in Chen and Hutchinson, 2004; Huang *et al.*, 2005). The relations between the wavelength and plate 1 (film) thickness that was computed using the model equations and our protocol are similar. For a large difference in thickness between plates 1 and 2, our protocol gave similar results to the model equations (row 6 in Table S1). However, the computed wavelength was the same for a range of small h_1 (rows 6-8) because we were searching for the minimum energy configurations within the wavenumber range, which means they have a low wavelength resolution. If the thickness of plate 1 was relatively large (rows 1-3) the results of the models and our protocol diverged more strongly, which can be explained by the fact that such assumptions on plate thickness are not applicable to the model equations. Therefore, our protocol though simplistic, gives results that are similar to the buckling models when the ranges of the thickness and stiffness of the components of the layered structure are similar. On the other hand, the protocol complements the models, thereby providing solutions for cases in which there is a relatively thick "film" and low difference in stiffness.

Supplementary Table S1. Wavelengths of buckled surfaces computed with the same assumptions using the computation protocol dedicated to cell walls and models on buckling of the layered structure (Chen and Hutchinson, 2004; Huang *et al.*, 2005).

Plate thickness [µm]		Computed wavelengths [µm]			
h_1	h_2	λ_{comp}	$\lambda_{model}\,1$ ($\nu = 0$)	$\lambda_{model}\,1$ ($\nu = 0.4$)	$\lambda_{model}\,2$ ($\nu = 0$)
0.5	1	0.698	7.982	6.891	13.722
0.2	1	0.331	4.014	3.466	5.489
0.1	1	0.203	2.387	2.061	2.744
0.07	1	0.179	1.827	1.577	1.921
0.05	1	0.170	1.419	1.225	1.372
0.01	1	0.170	0.424	0.366	0.274
0.001	1	0.170	0.075	0.065	0.027
0.0001	1	0.170	0.013	0.012	0.003

The plate 1 wavelength (λ_{comp}) was computed for various thickness of plate 1 using the computation protocol (*Supplementary Protocol S3*), assuming that E_1 = 10000 MPa; $E_2 = E_3$ = 40 MPa; $E_W = E_2$; $\sigma_{1,2,3}$ = 7.2 MPa. To account for the increased ranges of modulus and plate thickness, the minimal energy solutions were searched for within the wavenumber range five times wider than for the cell walls. Equation (18) from Huang *et al.* (2005) was used for computation of λ_{model} *1*; equation (17) for computation of λ_{model} *2*. The two values of Poisson ratio taken to compute λ_{model} *1* are the extreme values allowed for this equation. h_1 and h_2 – thickness of plate 1 and 2, respectively; v – Poisson ratio.
