# Supplementary material for: Growing cell walls show a gradient of elastic strain across their layers
Source: J Exp Bot. 2018 Jun 26;69(18):4349–62. doi: 10.1093/jxb/ery237 (PMC6093493; doi:10.1093/jxb/ery237)
Supplement: Supplementary Figures [file ery237_suppl_supplementary_figures_s1_s4.pdf]

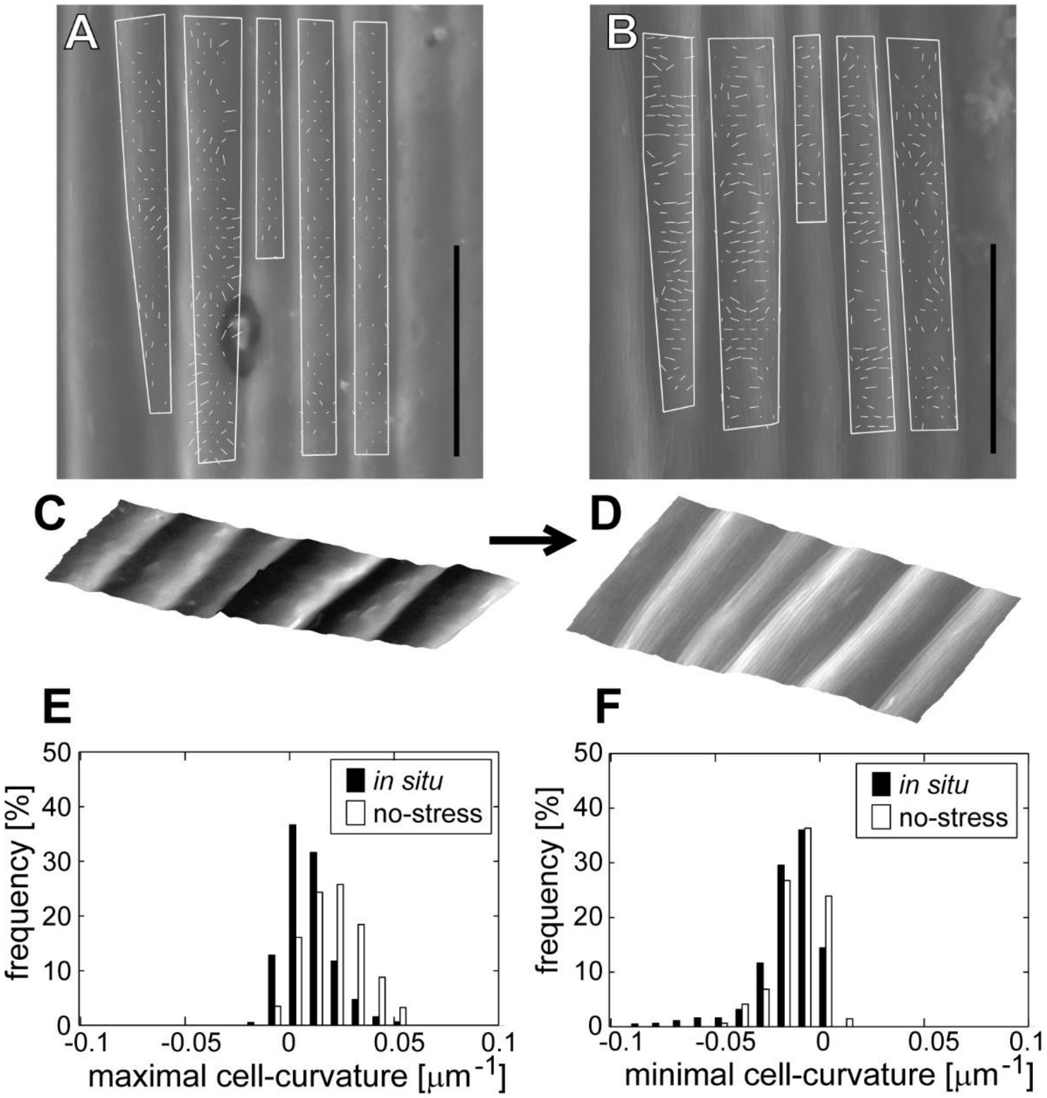

Supplementary Fig. S1 Changes in cell-curvature of the outer periclinal cell walls of epidermis of barley coleoptile due to the stress removal. Note that (C, D) show only portions of the regions shown in (A, B), which were chosen to illustrate the geometry of the reconstructed surface. Labelling as in Fig. 2. Bars, 50 μm.

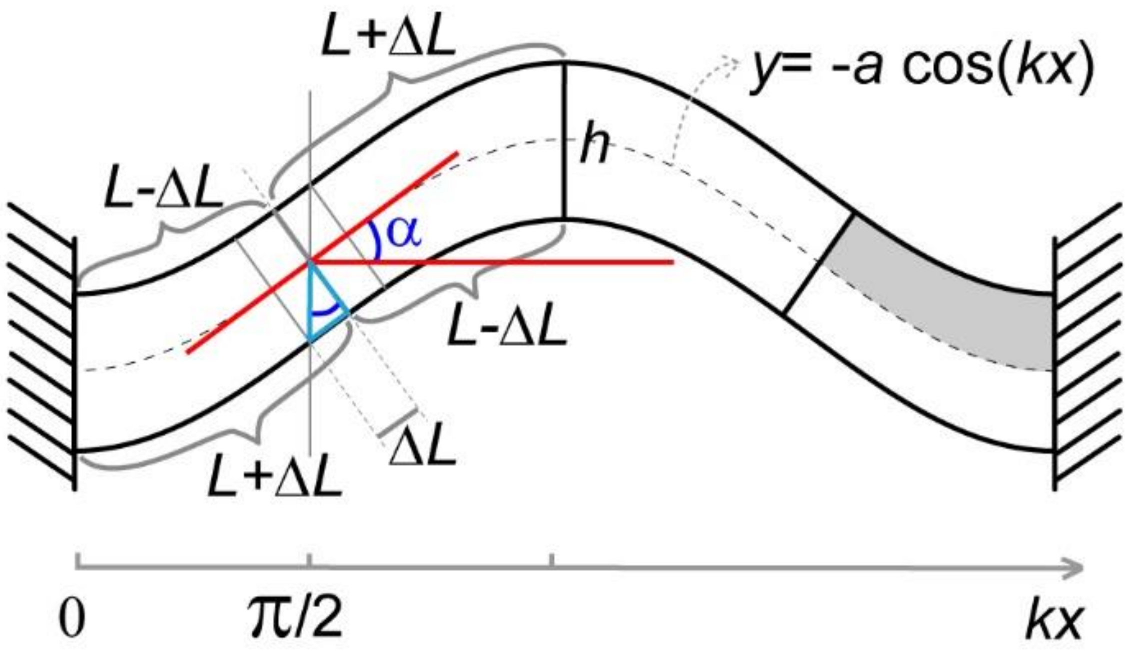

Supplementary Fig. S2 Estimation of plate deformation during buckling. The plate fragment shown in Fig. 6C is plotted in the scale *k*. The coloured line segments are used for calculation of *ΔL* as explained in *Supplementary Note S3*.

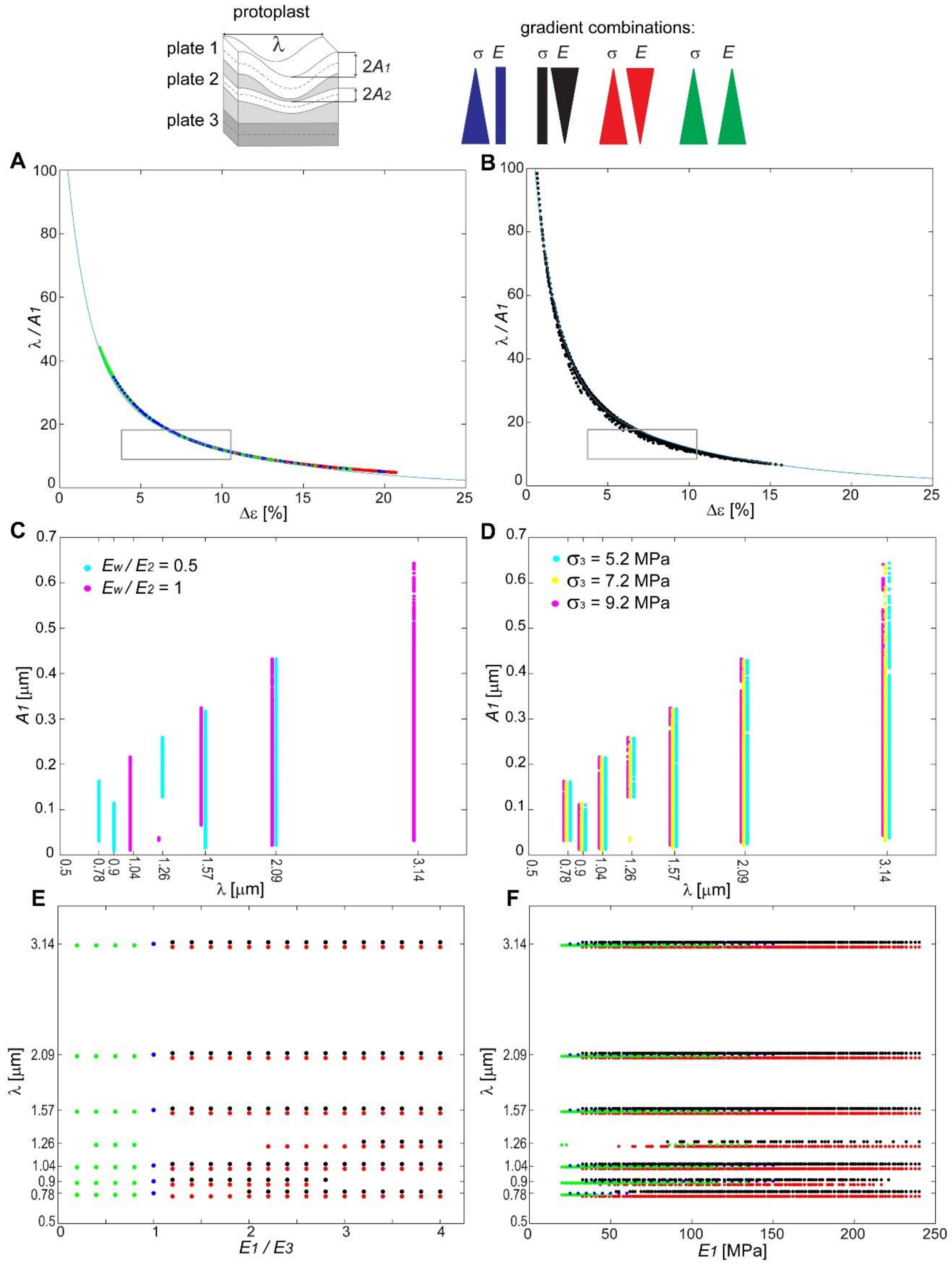

Supplementary Fig. S3 Minimum energy configurations of plate 1 computed for different mechanical parameters. (A, B) λ/A_1 is plotted against the difference in shrinking between plates 1 and 3 ($\Delta\varepsilon$), labelled by different colours for the four gradient combinations. Note that for better visibility the results for the "black" gradient are in a separate plot (B). The same exponential curve (cyan) is plotted in (A) and (B). The curve, given by the equation $y = 95.09e^{-0.7295x} + 35.78e^{-0.109x}$; fits to points for all the gradient combinations (R^2 = 0.996). Solutions within the range of empirical values are outlined in grey. (C, D) Amplitude and wavelength are plotted in different colours for different moduli of the elastic medium (C) and for different pre-stress of plate 3 (D). (E, F) Wavelength for different gradient combinations is plotted against the modulus ratio (E) or plate 1 modulus (F). In (C-F) points are offset in order to improve visibility.

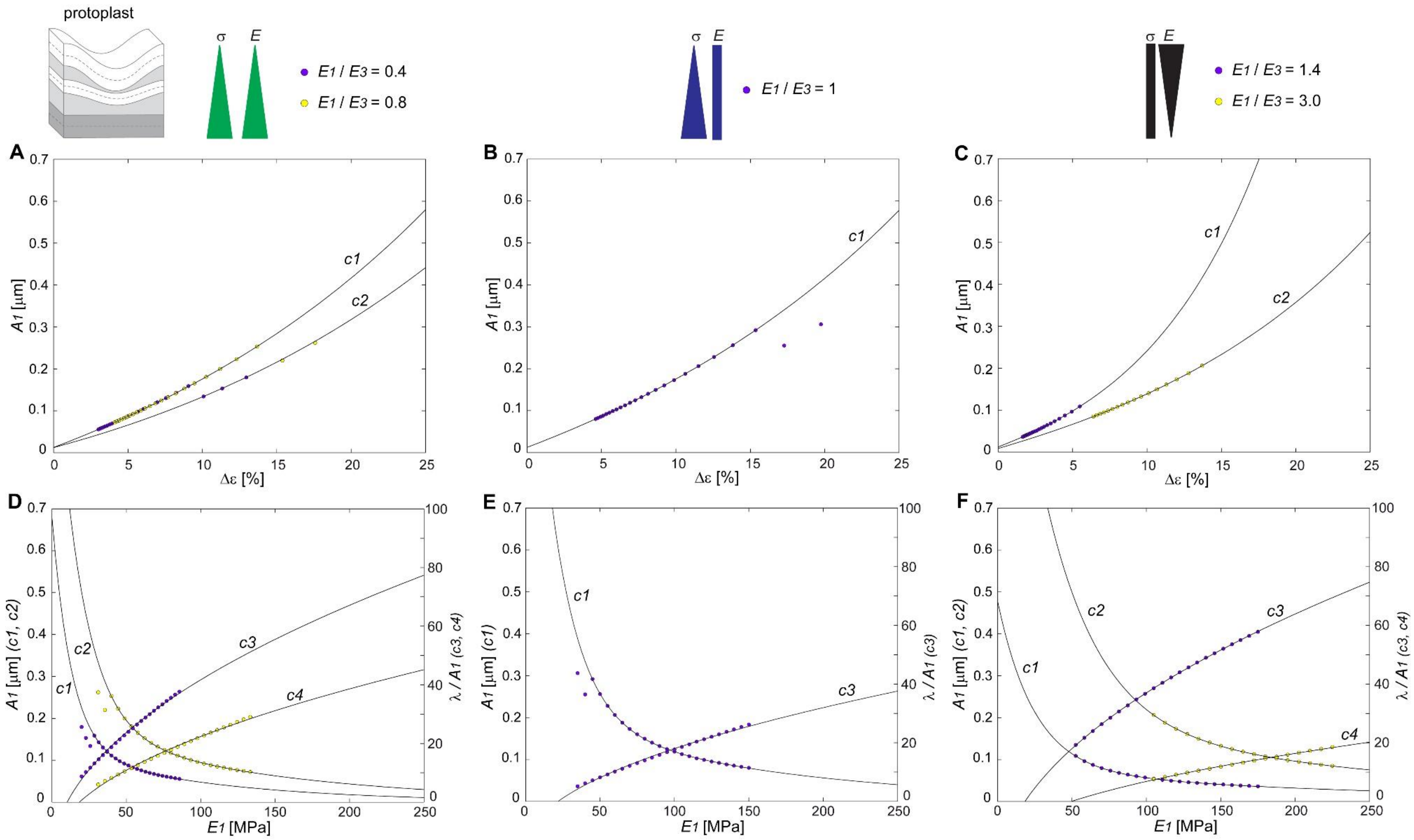

Supplementary Fig. S4 Response of buckled plate shapes to plate mechanics. Plots show relation between shape and mechanical parameters of plate 1, for minimum energy configurations computed for the aligned modulus and pre-stress gradients (A, D), outward pre-stress gradient (B, E), and inward modulus gradient (C, F). Dot colour represents the modulus ratio (E_1/E_3). All the plots show the minimum energy configurations for h_1 = 0.75 µm; σ_3 = 7.2 MPa; $E_W = 0.5E_2$. Curves *c1*, *c2* are given by the equation: $y = ae^{bx} + ce^{dx}$ (in A-C $a > 0$; $b < 1$; $c, d \in (-1,0)$; in D-F $a > 1$; $b, d \in (-1,0)$; $c \in (0,1)$). Curves *c3*, *c4* are given by: $y = a\sqrt{x} + b$ ($a > 1$; $b < 0$). In all the cases $R^2 > 0.9$. Note that amplitude values related to the different wavelengths fit to different curves.
